# Naringenin improves insulin sensitivity in gestational diabetes mellitus mice through AMPK

**DOI:** 10.1038/s41387-019-0095-8

**Published:** 2019-10-07

**Authors:** Sen Li, Yan Zhang, Yewu Sun, Guangzhen Zhang, Jie Bai, Jianfei Guo, Xudong Su, Hongquan Du, Xi Cao, Jinkui Yang, Ting Wang

**Affiliations:** 1 Department of Endocrinology, Liaocheng People’s Hospital, No. 67 Dongchang West Road, Liaocheng, 252000 Shandong China; 2 Department of Obstetrics and Gynecology, Liaocheng People’s Hospital, No. 67 Dongchang West Road, Liaocheng, 252000 Shandong China; 3 Department of Gynecology, the Affiliated Hospital of Qingdao University, No. 16 Jiangsu Road, Qingdao, 266000 Shandong China; 4 Department of Endocrinology, Beijing Tongren Hospital, Capital Medical University, No.1 Dongjiaominxiang Dongcheng District, Beijing, 100730 China; 5 Key Laboratory for Pediatrics of Integrated Traditional and Western Medicine, Liaocheng People’s Hospital, No. 67 Dongchang West Road, Liaocheng, 252000 Shandong China

**Keywords:** Biochemistry, Diseases

## Abstract

**Background:**

Gestational diabetes mellitus (GDM) is a temporary form of diabetes during pregnancy, which influences the health of maternal-child in clinical practice. It is still urgent to develop new effective treatment for GDM. Naringenin is a bioactive ingredient with multiple activities including anti-diabetic. In current study, the effects of naringenin on GDM symptoms, insulin tolerance, inflammation, and productive outcomes were evaluated and the underlying mechanisms were explored.

**Methods:**

We administrated naringenin to GDM mice and monitored the GDM symptoms, glucose and insulin tolerance, inflammation and productive outcomes. We established tumor necrosis factor alpha (TNF-α)-induced insulin resistance skeletal muscle cell model and evaluated the effects of naringenin on reactive oxygen species (ROS) production, glucose uptake and glucose transporter type 4 (GLUT4) membrane translocation.

**Results:**

We found that naringenin ameliorated GDM symptoms, improved glucose and insulin tolerance, inhibited inflammation, and improved productive outcomes. It was further found that naringenin inhibited TNF-α-induced ROS production, enhanced GLUT4 membrane translocation, and glucose uptake, which were abolished by inhibition of AMP-activated protein kinase (AMPK).

**Conclusion:**

Naringenin improves insulin sensitivity in gestational diabetes mellitus mice in an AMPK-dependent manner.

## Introduction

Gestational diabetes mellitus (GDM) is defined as glucose intolerance which is diagnosed during pregnancy. GDM is the most common metabolic condition during pregnancy and is associated with ~7% of all pregnancies^1^.

GDM manifests with symptoms including gestational hypertension, insulin resistance, fetal mal-development, and subclinical metabolic dysfunction. GDM results in short- and long-term health risks for mother, developing fetus and offspring, which include the subsequent maternal type 2 diabetes (T2DM), and possible adverse cardiometabolic phenotypes in the offspring. Decreased maternal insulin sensitivity or increased insulin resistance is implicated in GDM pathophysiology^2^. Insulin resistance results in decreased glucose uptake in skeletal muscle, white adipose tissue and liver, as well as decreased suppression of endogenous glucose production.

The major part of insulin-stimulated whole-body glucose is disposed in skeletal muscle, which plays an important role in the pathogenesis of insulin resistance. Insulin increases glucose uptake in skeletal muscle by activation of phspatidylinositol-3 kinase (PI3K), and Akt, resulting in increased translocation of glucose transporter type 4 (GLUT4) to plasma membrane. Insulin resistance in skeletal muscle leads to type 2 diabetes development^3^. It has been described that in GDM women, the insulin-stimulated glucose transport in skeletal muscle is markedly impaired. In addition, the decreased tyrosine phosphorylation of the insulin receptor β-subunit is associated with decreased glucose transport activity in GDM subjects^4^. Therefore, it could be useful for diabetes treatment by targeting skeletal muscle insulin sensitivity^5,6^.

Naringenin is one of the major citrus flavonoids predominantly found in grapes and oranges. Naringenin has been reported to have many pharmacological properties, including anti-inflammation^7^, cardioprotective^8^, anti-dyslipidemic^9^, anti-obesity and anti-diabetic^10^, and anti-fibrotic^11^. It has been shown that naringenin-stimulated glucose uptake in skeletal muscle cells in an AMP-activated protein kinase (AMPK) dependent manner. AMPK acts as an energy sensor and is activated by an increase in AMP/ATP ratio through phosphorylation^12^. Decreased AMPK activity is associated with insulin resistance while AMPK activation increases insulin sensitivity. As naringenin could stimulate glucose uptake in skeletal muscle and increase insulin sensitivity, we hypothesized that naringenin could also ameliorate the GDM syndrome. In current study, we aim to evaluate the effects of naringenin on GDM syndrome, skeletal muscle insulin sensitivity, and glucose uptake in a GDM mice model.

## Materials and methods

### Animals and study design

Six to eight weeks old C57BL/KsJ^+/+^ (wild type) and C57BLKsJ^db/+^ (db/+) mice were purchased from Jackson Laboratories (Bar Harbor, ME, USA) and used as genetic GDM model as indicated previously^13^. The mice were fed with chow diet containing 29% protein, 47% carbohydrate, and 17% fat (Envigo’s Teklad, USA). All mice were housed in a room with controlled temperature (22 °C), humidity (50%) and 12/12 h light cycle. For experimental design, female mice were randomly divided into three experimental groups: wild type, ad libitum fed; db/ + pair-fed, food intake of the ad libitum-fed wild type was measured daily, and the same amount of food was pair-fed to db/ + mice; db/ + pair-fed + Naringenin. A stock naringenin (Sigma-Aldrich, St. Louis, MO, USA) solution was prepared in 1% carboxymethylcellulose sodium (CMC) solution and administered to mice at a dose of 100 mg/kg b. w./day by oral gavage for 4 weeks as described previously^14^. Wild type and db/ + pair-fed group mice were administrated with equal volume of 1% CMC solution as control. At 10–12 weeks of age, the female mice were individually mated with males of the same genotype, and mating was confirmed by the presence of a copulatory plug the next morning, which was designated gestation day (GD) 0. Only the mice which became pregnant were used in each experimental group with 7–12 mice per group. All animal studies were approved by the Ethical Committee in Liaocheng People’s Hospital.

### Measurement of body weight, serum glucose

Maternal body weight and blood glucose were measured on GD0, 9, and 18 in wild type, db/+pair-fed and db/ + pair-fed + naringenin mice. Body weight was measured on a top-loading balance. Non-fasting blood sample were obtained via tail venipuncture and serum glucose level was measured using the glucometer (Roche Diagnostics, Risch-Rotkreuz, Switzerland).

### Glucose and insulin tolerance tests

On GD15, mice were fasted 6 h and injected intraperitoneally with 2.0 g/kg glucose for the glucose tolerance test. Then, blood glucose concentrations were analyzed using an ACCU-CHEK advantage glucometer (Roche Diagnostics). The glucose tolerance tests were recorded at 0, 30, 60, 90, and 120 min after glucose injection. For the insulin tolerance tests, mice were intraperitoneally injected with insulin (1.0 mU/kg) after a 60-min fast and the blood glucose concentrations were measured at baseline and after insulin injection (30, 60, 90, and 120 min). The homeostasis model assessment for insulin resistance (HOMA-IR) was calculated by (fasting blood glucose [mmol/l]*fasting plasma insulin [µU/ml])/22.5 as described previously^15^. Quantitative Insulin Sensitivity Check Index (QUICKI) was calculated by 1/(log fasting blood glucose [mmol/l] + log fasting plasma insulin [µU/ml]) as described previously^16^.

### ELISA

Plasma adiponectin were measured by ELISA on GD15 using commercial ELISA kits purchased from R&D systems (Minneapolis, MN, USA) following manufacture’s protocols. On GD18, serum levels of interleukin-1β (IL-1β), interleukin-6 (IL-6), tumor necrosis factor α (TNF-α), and monocyte chemotactic protein 1 (MCP-1) were measured by ELISA using commercial ELISA kits (R&D Systems).

### Western blot

Skeletal muscles including triceps, pectorals, and all limb muscles were harvested from each mouse, pooled and treated as individual samples. ReadyPrep™ Protein Extraction Kit (Bio-Rad, Hercules, CA, USA) was used to extract total skeletal muscle proteins. Protein concentration was measured using Pierce™ BCA Protein Assay Kit (Thermo Fisher, Waltham, MA, USA). Total 20 µg proteins were loaded onto 12% sodium dodecyl sulfate–polyacrylamide gel electrophoresis gel and transferred to polyvinylidene fluoride membrane. The membranes were blocked in 5% non-fat milk at room temperature for 1 h and then the incubated with primary antibodies overnight. Next day, membranes were washed with wash buffer (Thermo Fisher) for three times and then incubated with corresponding horse radish peroxidase-conjugated secondary antibodies at room temperature for 1 h. Primary antibodies used in current study were anti-TLR2 (Abcam, Cambridge, MA, USA), anti-TLR4 (Abcam), anti-phospho-JNK (Thermo Fisher), anti-JNK (Thermo Fisher), anti-phospho-NF-κB p65 (Abcam), anti-NF-κB p65 (Abcam), phospho-AMPK (Cell Signaling Technology, Beverly, MA, USA), AMPK (Cell Signaling Technology), and anti-β actin (Sigma). Clarity™ Western ECL Blotting Substrates (Bio-Rad) was used to detect the immunoreactive proteins. The density was quantitated using GS-900™ Calibrated Densitometer (Bio-Rad) and analyzed by using Image Lab (Bio-Rad). For certain experiment, the expression of each protein was normalized to β actin expression first, and then the expression of each protein in GDM and naringenin-treated GDM mice was normalized to the protein level in normal mice.

### Quantitative real-time polymerase chain reaction (PCR)

Total RNA from skeletal muscles was isolated using Trizol reagent (Thermo Fisher) following the manufacturer’s protocols. Then RNA was reverse-transcribed using SuperScript^®^ III First-Strand Synthesis System (Thermo Fisher). Real-time PCR was performed using QuantiTect SYBR Green PCR Kit (Qiagen, Germantown, MD, USA) on a QuantStudio 5 Real-Time PCR System (Thermo Fisher). The following primers were used in the current study: IL-1β, Forward 5′-AACCTGCTGGTGTGTGACGTTC-3′, Reverse 5′-CAGCACGAGGCTTTTTTGTTGT-3′; IL-6 Forward 5′-ACAACCACGGCCTTCCCTACTT-3′, Reverse 5′-CACGATTTCCCAGAGAACATGTG-3′; TNF-α Forward 5′-GCCTCTTCTCATTCCTGCTTG-3′, Reverse 5′-CTGATGAGAGGGAGGCCATT-3′; MCP-1 Forward 5′-CCACTCACCTGCTGCTACTCAT-3′, Reverse 5′-TGGTGATCCTCTTGTAGCTCTCC-3′; β actin Forward 5′-CGTGCGTGACATCAAAGAGAA-3′, Reverse 5′-TGGATGCCACAGGATTCCAT-3′. The amount mRNA expression was normalized with β actin mRNA value first, and then mRNA expression of each protein in GDM and naringenin-treated GDM mice was normalized to the mRNA level in normal mice.

### Fetal outcome analysis

Pregnant mice were anesthetized by ketamine–xylazine and then euthanized on GD18 by cardiac puncture with the heart cut. After performing Cesarean section, the litter size was counted in combination with their location along the length of the respective uterine horn. Viable fetuses were identified by virtue of their ability to move and breathe, and weighed.

### Cell culture and treatment

C2Cl2 mouse myoblasts were obtained from American Type Culture Collection (Manassas, Virginia, USA) and maintained in DMEM supplemented with 10% fetal bovine serum (Thermo Fisher) in an incubator containing 5% at 37 °C. Myoblast differentiation was induced with DMEM supplemented with 5% horse serum for 72 h.

### Reactive oxygen species (ROS) detection

C2Cl2 cells were plated on coverslips in 24-well plate and cultured for overnight. The cells were treated with 2 ng/ml TNF-α for 36 h, together with or without 50 μg/mL naringenin. Before harvesting, cells were treated with 4 µM dorsomorpin (Sigma) for 1 h. DCFH-DA staining was used to measure ROS production. Briefly, cells were washed with phosphate-buffered saline (PBS) and 200 µl DCFH-DA (10 µM) (Sigma) was added to cells. After 30 min incubation at 37 °C in dark, cells were washed with PBS and the intracellular ROS production was measured under microscope. Then the fluorescence intensity within each cell was quantified by ImageJ.

### GLUT4 detection

Cells were pre-treated with 2 ng/ml TNF-α for 36 h, together with or without 50 μg/mL naringenin. Cells were further treated with 4 µM dorsomorpin for 1 h and then serum starved for 4 h. After serum starvation, cells were then stimulated with 10 nM insulin for 10 min at 37 °C. Then cells were sonicated and lawns were fixed, blocked and labeled with rabbit anti-GLUT4 antibody at room temperature for 1 h. After wash, Alexa Fluor 488 conjugated secondary antibody was added and incubated for 45 min. Lawns were outlined and fluorescence intensity was measured by microscope and analyzed by ImageJ.

### 2-Deoxyglucose uptake

2-Deoxyglucose uptake was measured using Glucose uptake assay kit (Abcam) following manufacturer’s protocol. Briefly, cells were pre-treated with 2 ng/ml TNF-α for 36 h, together with or without 50 μg/mL naringenin. Cells were further treated with 4 µM dorsomorpin for 1 h and then serum starved for 4 h. After serum starvation, cells were then stimulated with 10 nM insulin for 10 min at 37 °C. Then 2-DG was added to cells and incubated for 20 min at 37 °C. After washing, cells were lysed and supernatants were tested.

### Statistical analysis

All data were presented as mean ± standard deviation (SD). Data were analyzed by one or two-way ANOVA test followed by Tukey’s post hoc test. The statistical difference was considered as significant when *p* value is <0.05.

## Results

### Naringenin treatment ameliorates diabetes mellitus symptoms in GDM mice

**Fig. 1:**
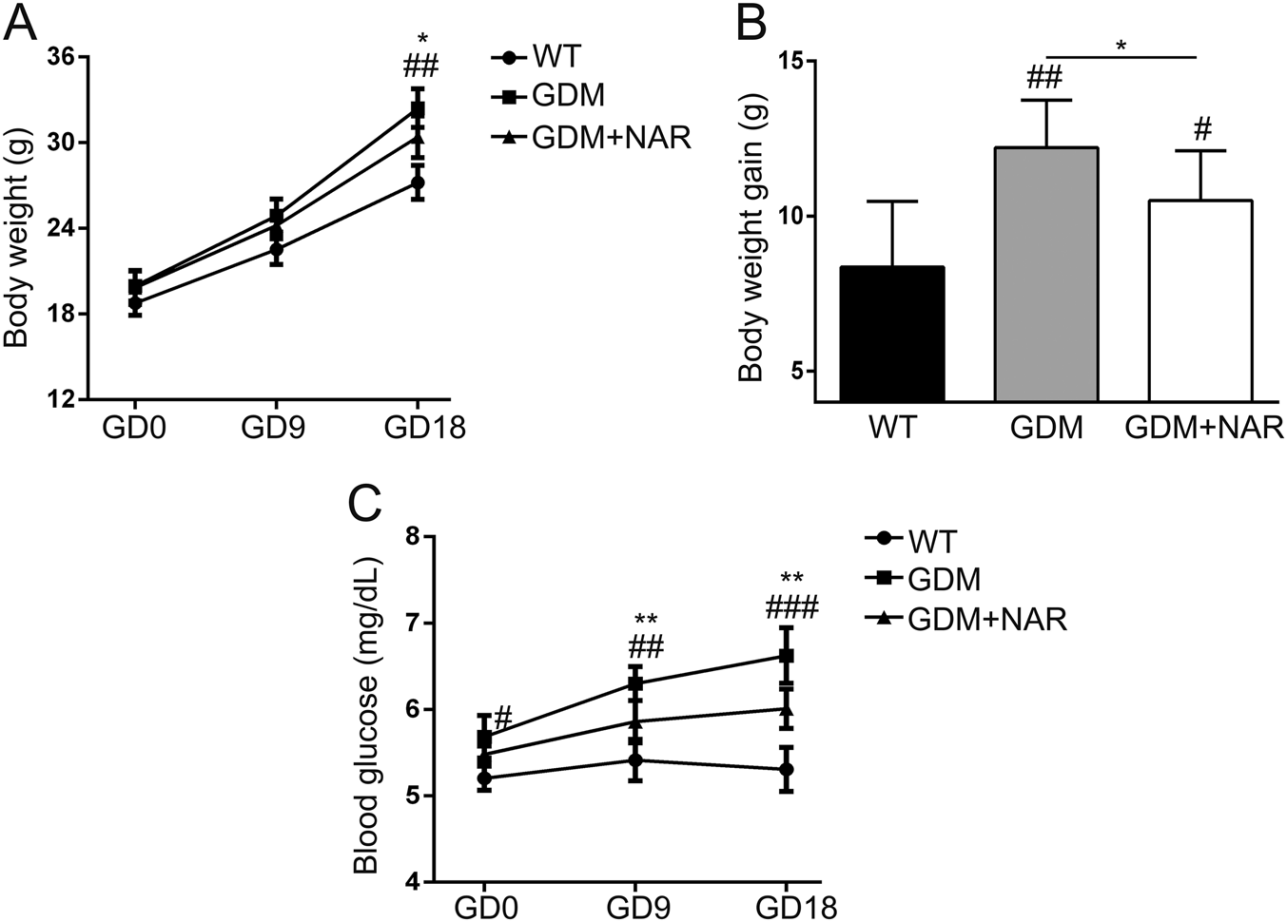
Naringenin administration alleviates gestational diabetes mellitus (GDM) symptoms. **a** Maternal body weight was recorded on gestation day (GD) 0, 9, and 18 in wild-type group, GDM group, and GDM + NAR group. **b** Body weight gains were calculated from GD0 to GD18. **c** Serum glucose levels of each group were measured on GD0, 9, and 18. NAR, naringenin for short. *n* = 7–12 for each group. Data were presented as mean ± SD. ^#^*p* < 0.05, ^##^*p* < 0.01, ^###^*p* < 0.001 compared with wild-type group. *p < 0.05, **p < 0.01 between the comparison of GDM group and GDM + NAR group

First, we evaluated the effects of naringenin on body weight and blood glucose level of GDM mice throughout the pregnancy. As shown in Fig. 1a, all three groups of mice had increased body weight during pregnancy. Although there was no significantly difference of body weight among all groups on GD0 and GD9, the body weight of GDM mice was significantly higher than that of normal mice on GD18. In contrast, naringenin-treated GDM mice had significantly lower body weight than non-treated GDM mice on GD18, indicating that naringenin treatment prevented the increasing of body weight in GDM mice. However, naringenin-treated GDM mice still had significantly higher body weight than normal mice, indicating that naringenin cannot normalize the body weight of GDM mice to that of wild-type/normal mice. Correspondingly, GDM mice gained significantly more body weight from D0 to D18 than wild-type/normal mice. The body weight gain of naringenin-treated GDM mice was significantly less than that of non-treated GDM mice, while was still significantly more than that of wild-type/normal mice (Fig. 1b). The serum glucose levels of normal pregnancy wild-type mice remained stable on GD0, 9, and 18 (Fig. 1c). In contrast, GDM mice showed significantly increased blood glucose levels at GD0, GD9, and GD18 when compared with wild-type mice. In contrast, naringenin treatment resulted in significantly decreased blood glucose levels in GDM mice on GD9 and GD18. Interestingly, naringenin treatment did not normalize the serum glucose level of GDM mice to that of wild-type mice. Taken together, our data demonstrated that naringenin ameliorated but did not normalize diabetes mellitus symptoms in GDM mice.

### Naringenin supplementation improves glucose and insulin tolerance in GDM mice

**Fig. 2:**
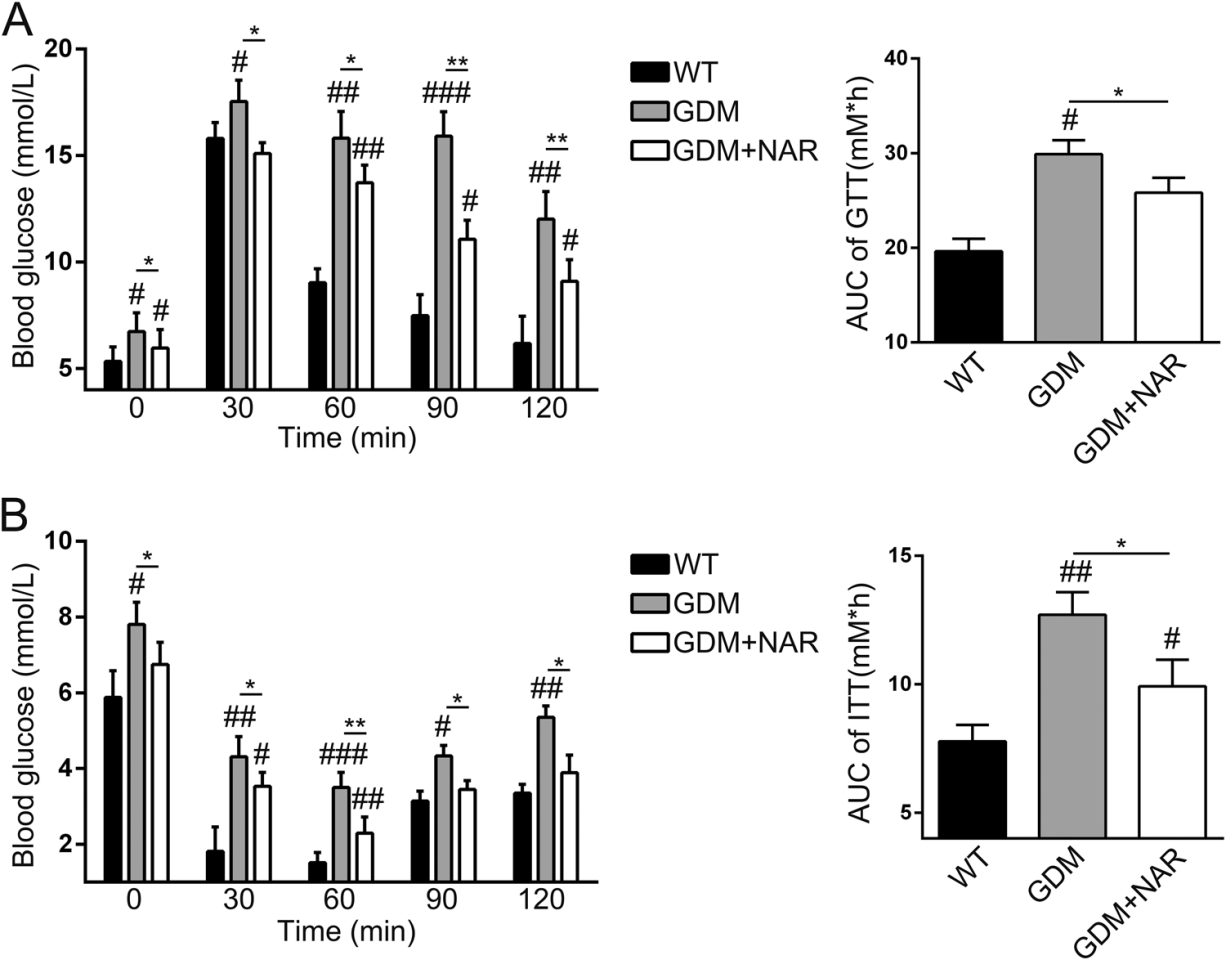
Naringenin supplementation improves glucose and insulin tolerance in GDM mice. The glucose tolerance test (GTT) and insulin tolerance test (ITT) were performed on gestation day (GD) 15. **a** Effect of naringenin on glucose tolerance on GD15 in GDM mice. **b** Effect of naringenin on insulin tolerance on GD15 in GDM mice. *N* = 7–12 for each group. Data were presented as mean ± SD. ^#^*p* < 0.05, ^##^*p* < 0.01, ^###^*p* < 0.001 compared with wild-type group. *p < 0.05, **p < 0.01 between the comparison of GDM group and GDM + NAR group

We continued to evaluate the effects of naringenin on glucose and insulin tolerance in GDM mice. Consistent to the data presented in Fig. 1, on GD15 and before glucose injection, the blood glucose level of GDM mice was significantly increased when compared with that of wild-type mice (Fig. 2a). The naringenin treatment significantly decreased the blood glucose level in GDM mice. After injection of glucose, the blood glucose level of all three groups increased. The blood glucose levels of GDM mice were significantly higher than that of wild-type mice at all four time points (30, 60, 90, and 120 min) post glucose injection. Naringenin significantly decreased the blood glucose level in GDM mice. However, the blood glucose levels of naringenin-treated GDM mice were significantly higher than that of wild-type mice after glucose injection. Correspondingly, the glucose area under the curve (AUC) of GDM mice was significantly larger than that of wild-type mice. In contrast, naringenin-treated GDM mice had greatly smaller AUC than non-treated GDM mice. Therefore, our data demonstrated that naringenin improved glucose tolerance in GDM mice. Injection of insulin resulted in decreased blood glucose levels in all three groups of mice. The blood glucose levels of GDM mice were significantly higher than that of wild-type mice at 30, 60, 90, and 120 min after insulin injection (Fig. 2b). In contrast, naringenin treatment significantly decreased the blood glucose levels in GDM mice at all four time points. However, we still detected that the blood glucose levels of naringenin-treated GDM mice were significantly higher than that of wild-type mice. Correspondingly, glucose area under the curve (AUC) of naringenin-treated GDM mice was significantly lower than that of GDM mice, while was significantly higher than that of wild-type mice. Naringenin treatment also significantly decreased fasting blood insulin level and HOMA-IR in GDM mice, although failed to normalize them to these in wild-type mice (Table 1). Naringenin treatment significantly decreased and normalized fasting blood glucose level in GDM mice to that in wild-type mice. The QUICKI value was significantly decreased in GDM mice when compared with wild-type mice. In contrast, naringenin treatment significantly increased QUICKI value in GDM mice to similar level in wild-type mice, indicating that naringenin enhanced the insulin sensitivity in GDM mice. Similarly, naringenin treatment significantly increased the blood level of adiponectin, the endogenous insulin sensitizer, in GDM mice (Table 1). Taken together, our data demonstrated that naringenin improved glucose and insulin tolerance in GDM mice.

**Table 1:** Levels of fasting blood glucose, insulin sensitivity indices and adiponectin of maternal mice from different groups on GD15

Parameters	WT (*n* = 8)	GDM (*n* = 11)	GDM + NAR (*n* = 10)
Insulin (ng/mL)	0.61 ± 0.08	0.79 ± 0.11^a,b^	0.75 ± 0.12^a^
Fasting blood glucose (mmol/L)	3.97 ± 0.71	6.84 ± 1.03^a,b^	4.38 ± 0.89
HOMA-IR	4.01 ± 0.17	9.52 ± 0.31^a,b^	6.12 ± 0.23^a^
QUICKI	0.424 ± 0.03	0.27 ± 0.01^a,b^	0.378 ± 0.02
Adiponectin (μg/mL)	6.56 ± 1.24	3.47 ± 1.86^a,b^	5.61 ± 1.63

Notes: Data were presented as mean ± SD

^a^*p* < 0.05 vs wild-type group

^b^*p* < 0.05 between the comparison of GDM group and GDM + NAR group

### Naringenin inhibits inflammation in GDM mice

**Fig. 3:**
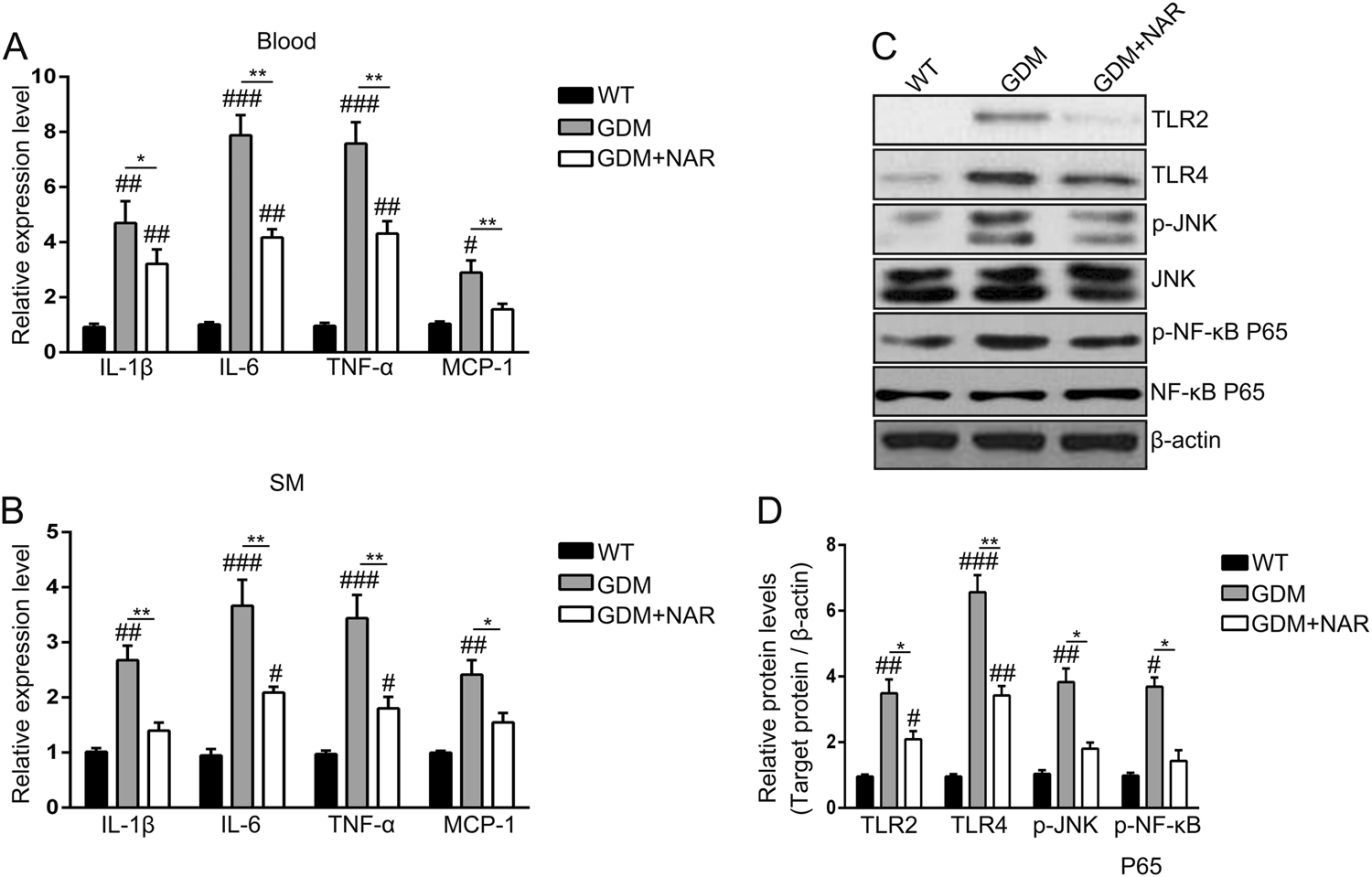
Naringenin inhibits inflammation in GDM mice. Relative expression levels of IL-1β, IL-6, TNF-α, and MCP-1 in mice blood (**a**) and SM (**b**) of different groups. SM, skeletal muscle; IL-1β, interleukin-1β; IL-6, interleukin-6; TNF-α, tumor necrosis factor α; MCP-1, monocyte chemotactic protein 1. *n* = 7–12 for each group. ^#^*p* < 0.05, ^##^*p* < 0.01, ^###^*p* < 0.001 compared with wild-type group. *p < 0.05, **p < 0.01 between the comparison of GDM group and GDM + NAR group. **c** Total cell lysates were extracted, and the amount of TLR2, TLR4, phospho-NF-κB p65, phospho-JNK and the corresponding total protein levels were analyzed by Western blotting. β-actin served as a loading control. Shown are representative results from three independent experiments. **d** Signals of proteins were normalized against β-actin and quantified and are presented as fold change of GDM groups vs wild-type group. Shown are means ± SD of three independent experiments. ^#^*p* < 0.05, ^##^*p* < 0.01, ^###^*p* < 0.001 compared with wild-type group. *p < 0.05, **p < 0.01 between the comparison of GDM group and GDM + NAR group

To evaluate the effects of naringenin on inflammation in GDM mice, firstly we monitored the blood levels of inflammatory cytokines. As shown in Fig. 3a. GDM mice had significantly elevated blood levels of IL-1β, IL-6, TNF-α, and MCP-1 when compared with wild-type mice. In contrast, the blood levels of these cytokines of naringenin-treated GDM mice were significantly decreased when compared with non-treated GDM mice. However, although naringenin decreased the cytokines levels in GDM mice, it cannot normalize the cytokines levels to these of wild-type mice, as we still detected significantly higher cytokines levels in naringenin-treated GDM mice when compared with wild-type mice. Similar cytokine profiles were detected in the skeletal muscle. As shown in Fig. 3b, we detected significantly increased expression of IL-1β, IL-6, TNF-α, and MCP-1in skeletal muscle of GDM mice when compared with wild-type mice. In contrast, naringenin treatment significantly decreased levels of these cytokines in GDM mice were decreased, while it cannot normalize the cytokine levels to that in wild-type mice. Besides inflammatory cytokines, signaling pathways factors involved in inflammation, including pattern recognition receptors (PPR) TLR2 and TLR4, phorspho-JNK and phorspho-NF-κB p65, were increased in skeletal muscle of GDM mice too (Fig. 3c, d). The protein levels of these factors were significantly decreased by naringenin treatment in GDM mice. In addition, the naringenin treatment normalized phorspho-JNK and phorspho-NF-κB p65 levels of GDM mice to these of wild-type mice, indicating naringenin efficiently prevented the activation of NF-κB and MAPK signaling pathway. Collectively, our data demonstrated that naringenin inhibited inflammation in GDM mice.

### Naringenin improved fetal outcomes in GDM mice

**Fig. 4:**
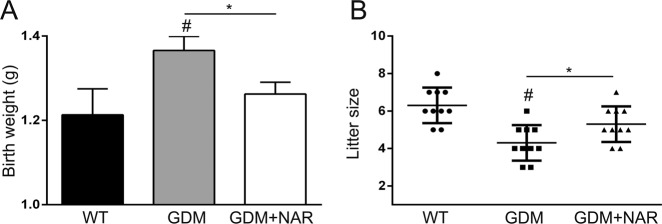
Naringenin improves the reproductive outcome of GDM mice. Body weight at birth (**a**) and litter size (**b**) of offspring born by each female mouse from different groups were recorded. *N* = 7–12 for each group. Data were presented as mean ± SD. ^#^*p* < 0.05, compared with wild-type group. *p < 0.05 between the comparison of GDM group and GDM + NAR group

Next, we explored the potential effect of naringenin on fetal outcomes in GDM mice. GDM mice showed significantly increased birth weight (Fig. 4a) and decreased litter size (Fig. 4b). Naringenin treatment significantly decreased birth weight and increased litter size in GDM mice. Taken together, our data demonstrated that naringenin improved fetal outcomes in GDM mice.

### Naringenin restored TNF-α-induced insulin resistance via AMPK

**Fig. 5:**
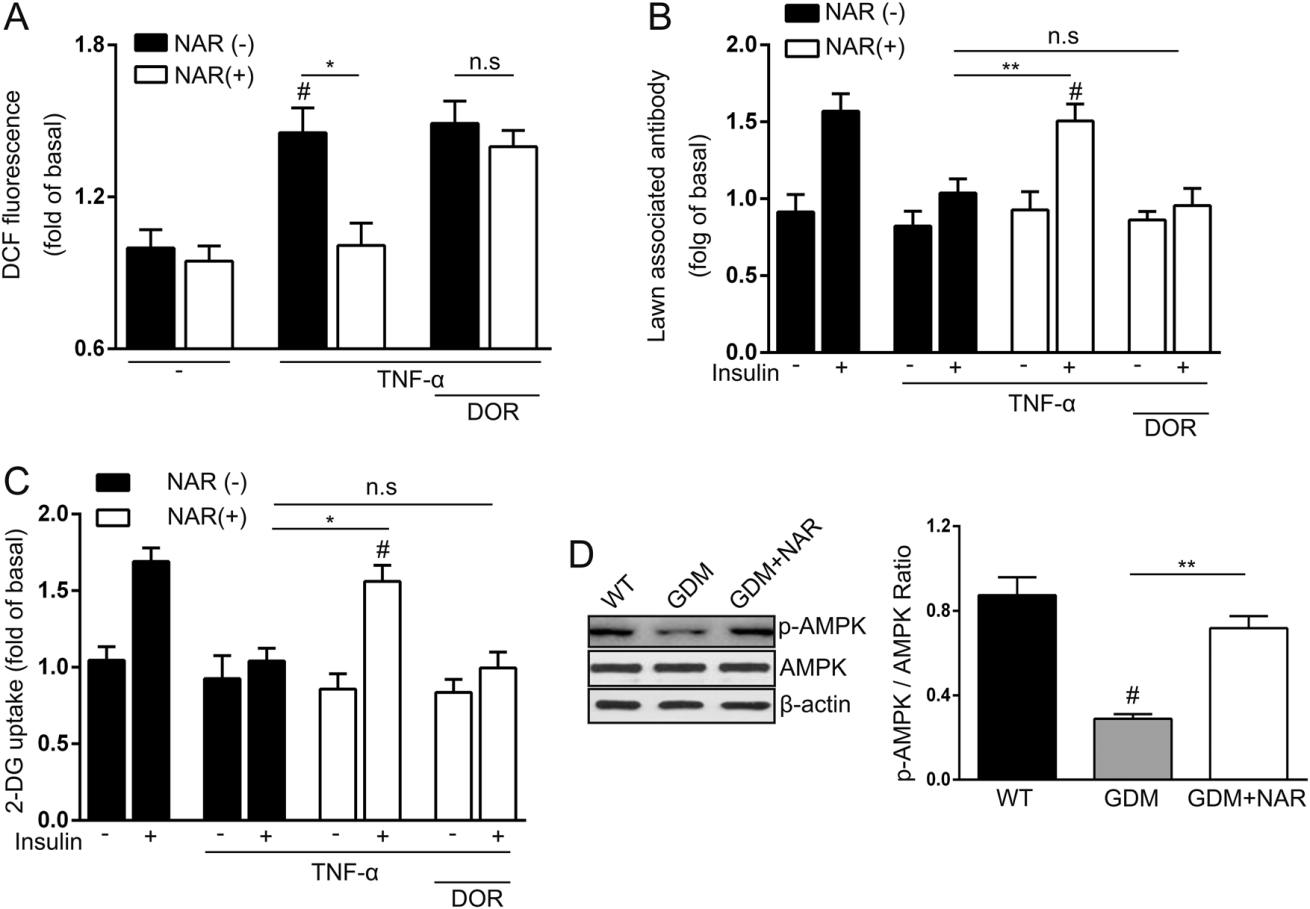
Naringenin restores TNF-α-induced insulin resistance through activation of AMPK. **a** Generation of ROS was monitored using 5 M DCFH-DA. Naringenin decreases ROS generated by TNF-α, which was abolished by dorsomorphin hydrochloride treatment. **b** Naringenin promoted the translocation of GLUT4 to the plasma membrane, which can also be attenuated by dorsomorphin hydrochloride administration. **c** The effect of Naringenin on promoting glucose uptake was abolished by orsomorphin hydrochloride treatment. DOR, dorsomorphin hydrochloride. All results performed above are presented as mean ± SD. *n* = 6. ^#^*p* < 0.05 compared with untreated cells stimulated by insulin at the same dose. *p < 0.05, **p < 0.01. n.s, not significant. **d** Western blot was performed to detect the active AMPK (p-AMPK) and total AMPK in the SM of different groups of mice. The p-AMPK /AMPK ration was also calculated. Shown are representative results from three independent experiments. ^#^*p* < 0.05 compared with wild-type group. **p < 0.01 between the comparison of GDM group and GDM + NAR group

Finally, we explored the underlying mechanism of naringenin-mediated protection on GDM. SM is the most important organ for whole-body glucose homeostasis and has been implicated in insulin resistance^17–19^. As naringenin has been described to increase muscle cell glucose uptake in an AMPK-dependent manner^20^, we hypothesized that naringenin may also utilized this activity for its protection in GDM mice. To test our hypothesis, we established the TNF-α-induced insulin resistance cell model^21^. As TNF-α has been shown to induce ROS production in this cell model, first we first evaluate the effects of naringenin on TNF-α-induced ROS production. As shown in Fig. 5a, at steady-state, the naringenin treatment did not affect endogenous ROS level. Once the cells were treated with TNF-α, the endogenous ROS level was significantly increased. In contrast, naringenin significantly decreased ROS level in TNF-α-treated C2C12 cells, indicating naringenin blocked TNF-α induced upregulation of ROS. Interestingly, this effect depended on AMPK, as inhibition of AMPK by inhibitor dorsomorphin hydrochloride rescued TNF-α-induced ROS production in naringenin -treated cell. Administration of insulin resulted in plasma membrane translocation of Glucose transporter type 4 (GLUT4) and TNF-α could inhibit the GLUT4 membrane translocation induced by insulin (Fig. 5b). Naringenin significantly increased GLUT4 membrane translocation in TNF-α-treated C2C12 cells, which also depended on AMPK activity, as inhibition of AMPK by dorsomorphin hydrochloride abolished the naringenin-medicated rescuing of insulin-induced GLUT4 membrane translocation in TNF-α-treated C2C12 cells. Similarly, naringenin also rescued glucose uptake in TNF-α-treated cells, which required AMPK activity too (Fig. 5c). All these data from the cell model suggested a very important role of AMPK in naringenin-mediated insulin sensitivity. Correspondingly, we detected significantly decreased phosphor-AMPK level in SM of GDM mice (Fig. 5d). In contrast, naringenin-treated GDM mice had significantly increased phosphor-AMPK level when compared with non-treated GDM mice. Taken together, our data demonstrated that naringenin ameliorated TNF-α-induced insulin resistance in an AMPK-depended manner.

## Discussion

GDM is a temporary form of diabetes during pregnancy. GDM is associated with increased fetal-maternal morbidity and the offspring of women with GDM are more likely to develop diabetes and metabolic syndrome. Currently the available strategies to treat women with GDM are limited. Insulin is the major treatment while is associated with hypoglycaemia and adverse placental, fetal and maternal outcomes^22^. Metformin, although is effective to treat GDM, can cross the placenta with unknown long-term effects. Metformin also caused gastrointestinal discomfort^23,24^. Therefore, searching for effective agents with limited side effects is in great demand.

Naringenin is a flavonoid found in citrus fruit and tomatoes that has been reported to provide protection in multiple aspects in diabetes^25–27^. These activities strongly suggested potential protective role of naringenin in GDM. In current study, the therapeutic effects of naringenin on GDM symptoms (maternal body weight and serum glucose), glucose and insulin tolerance were evaluated using GDM mice model. We demonstrated that naringenin alleviated GDM symptoms in GDM mice by significantly decreasing the body weight and blood glucose level. Naringenin also improved glucose and insulin tolerance. In addition, naringenin enhanced fetal outcomes in GDM mice by decreasing the birth weight and increasing litter size. All these results indicated that naringenin provided the protective effects on GDM, suggesting naringenin could be used as a potential therapeutic agent to treat GDM.

Maternal obesity and excessive gestational weight gain (GWG) are associated GDM. In current study, we found naringenin prevented the body weight increasing in GDM mice. We did not find obvious changes of food intake in GDM mice after naringenin treatment, which was consistent to previous reports demonstrating that administration of naringenin did not affect food intake^28^. In contrast, Burke et al. demonstrated that naringenin administration enhanced the energy expenditure and reduced adiposity in Ldlr^−/−^ mice^29^. These published findings could explain why naringenin decreased the body weight in GDM mice. In addition, the suppressed body weight gain in GDM mice after naringenin treatment also suggested improved insulin sensitivity.

Inflammation has been complicated in GDM and is associated increased maternal insulin resistance^30^. Elevated circulating levels of IL-6 and MCP-1 in maternal serum have been consistently observed in GDM^31^. Greater amounts of TNF-α were released from GDM patients’ tissues, which was supposed to be a predictor of insulin resistance^32,33^. In current study, we confirmed significantly elevated IL-6, IL-1β, TNF-α, and MCP-1 level in GDM mice. The TLR2 and TLR4 signaling pathways were also found to be activated in GDM mice. The anti-inflammation activity of naringenin has been well-studied in animal models. Tsai and colleagues described that in diabetic mice model, naringenin reduced renal TNF-α, IL-1β, IL-6, and MCP-1 level^11^. Naringenin also inhibited NF-κB activation and attenuated diabetic nephropathy in diabetic mice. Our current study also demonstrated that naringenin reduced the inflammatory levels in serum and skeletal muscle, and inhibited activations of NF-κB and MAPK signaling pathways.

Increased maternal skeletal muscle insulin resistance is a central feature of GDM pregnancies, which is responsible for increased fetal nutrient supply and finally leads to increased fetal adiposity^4,34^. Studies have shown that the insulin signaling pathway and glucose uptake in skeletal muscle from pregnant women are significantly impaired by pro-inflammatory cytokines TNF-α and IL-1β, and also by LPS and poly(I:C)^35^. A number of studies have described the direct role for TNF-α in the pathophysiology of insulin resistance. For example, Rui et al. demonstrated that TNF-α downregulated insulin receptor signaling in cultured skeletal muscle^36^. Furthermore, increased TNF-α was associated with insulin resistance in a broad range of conditions including aging, sepsis and obesity. Therefore, elevated TNF-α level in gestation could attenuate insulin signaling and decrease insulin sensitivity in GDM. Consistent to previous findings, we demonstrated that the increased production of TNF-α in skeletal muscle contributed to the decreased insulin sensitivity in GDM mice, while naringenin inhibited TNF-α production and improved insulin sensitivity in GDM mice. In addition, in the TNF-α-induced insulin resistant cell model, we also found naringenin restored TNF-α-induced insulin resistance.

AMP-activated protein kinase (AMPK) is a key metabolic enzyme which regulates glucose metabolism. Exercise, ex vivo contraction could activate AMPK and stimulate glucose uptake into skeletal muscle. The glucose uptake is associated with increased translocation of GLUT4 glucose transporter to the plasma membrane. In current study, we found that the amelioration of insulin resistance in skeletal muscle cells by naringenin was abolished by inhibiting AMPK. The upregulation of glucose uptake, GLUT4 membrane translocation by naringenin depended on AMPK. Consistently, in the animal model, AMPK activation was impaired in GDM mice, which could contribute to the insulin resistance in GDM mice. Naringenin treatment increased the AMPK activation, which could result in enhanced glucose uptake and decreased insulin resistance. Our finding was consistent to the finding presented by Zygmunt^20^. They described that naringenin increased AMPK activation and stimulated glucose uptake. Silencing of AMPK abolished naringenin-stimulated glucose uptake.

Therefore, our findings demonstrated that naringenin displayed protective effect in GDM mice by suppressing inflammation, ameliorated insulin and glucose tolerance, and improved fetal outcomes in GDM mice, suggesting that naringenin could be used as therapeutic agent to ameliorated GDM symptoms. In addition, by using the cell model, we demonstrated that the protective effects of naringenin depended on AMPK. Another important point we would point out is that although naringenin ameliorated GDM symptoms, it did not totally prevent or abolish them as GDM mice still got higher levels of inflammation, insulin resistance when compared with normal mice. Looking for therapeutic reagents which can prevent or cure GDM is still in urgent demand.

Our current study demonstrated the protective effects of naringenin on GDM, several limitations should be addressed in future study. The first is the safety of naringenin. Unfortunately the study about the naringenin safety is limited. Although there was no obvious side effect of naringenin described in animal model, the related clinical trial of naringenin safety was limited. More safety studies need to be carried out. Another concern is the animal model of GDM for testing. The C57BLKsJ^db/+^ (db/+) mice model is the classic model to study GDM. Besides this model, streptozotocin (STZ) model, high-fat diet (HFD) model were also widely used^37^. It would be useful to test the effects of naringenin using other GDM models.

Naringenin displayed protective effect in GDM mice and suppressed inflammation, restored insulin sensitivity in GDM mice. Naringenin could be utilized as therapeutic treatment for GDM.
